# The Global Road Traffic Death Rate and Human Development Index from 2000 to 2019: A Trend Analysis

**DOI:** 10.34172/aim.2024.18

**Published:** 2024-03-01

**Authors:** Mohammad Sayari, Mohammad Reza Rahmanian Haghighi, Kamran Bagheri Lankarani, Sulmaz Ghahramani, Behnam Honarvar

**Affiliations:** ^1^Health Policy Research Center, Institute of Health, Shiraz University of Medical Sciences, Shiraz, Iran; ^2^University of Nicosia Medical School, Nicosia, Cyprus; ^3^Erasmus JMD Program, Unit for Research in Emergency and Disaster, University of Oviedo, Oviedo, Spain

**Keywords:** Education, Human development index, Income, Life expectancy, Road traffic death rate

## Abstract

**Background:**

Numerous studies on the association between the human development index (HDI) and road traffic death rate (RTDR) merely focus on developed countries, not reflecting the relationship between the HDI components and RTDR in a time-trend analysis. Accordingly, this study analyzes the trends of RTDR and their association with the HDI and its components from 2000 to 2019.

**Methods:**

The RTDR data of 154 countries were imported into the unconditional latent growth model (LGM) to assess the RTDR trends. The impact of the HDI and its components (viz., education, income, and life expectancy [LE viz]) on the trajectory of RTDR was also evaluated using the conditional LGM.

**Results:**

The results of the unconditional LGM indicated an overall decreasing trend in RTDR. The conditional LGM results revealed the negative effect of the HDI and its components on the model parameters. The findings of random forests indicated that education and LE were the most crucial variables.

**Conclusion:**

Overall, this study emphasizes the significance of HDI and its components, particularly education and LE, in lowering the number of traffic fatalities. In this sense, improving formal education and LE could be one of the main policies that policymakers could consider to reduce RTDR.

## Introduction

 Road traffic death (RTD) is the eighth leading cause of mortality among all people and the first cause of mortality in children and youngsters.^1^ From 2007 to 2013, the number of RTD remained unchanged.^2^ Africa and Southeast Asia regions face road traffic death rates (RTDRs) higher than the global average, while such values in Europe and America have been the lowest among the World Health Organization (WHO) regions.^1^

 According to the United Nations Development Programme (UNDP), gross national income (GNI) per capita per se cannot be sufficient to assess development in different countries. Hence, education and health status should be taken into account.^3^ Therefore, since 1990, various measurement tools have been developed to assess development among various nations, including the human development index (HDI) and inequality-adjusted HDI (IHDI). The HDI consists of three dimensions, namely life expectancy (LE, years), education (years), and income as a standard of living (i.e., GNI per capita 2017 at the purchasing power parity [PPP] $).^4^ South Asia, East Asia, the Pacific, and Sub-Saharan Africa regions have accordingly had the most rapid growth in the HDI between 1990 and 2017. The Organisation for Economic Co-operation and Development (OECD) member countries have also experienced the least significant progress in HDI during this period.^3^

 The relationship between RTDR and the HDI is not the same between countries with an HDI of lower than 0.55 and those with higher HDI.^5^ The association between RTDR and social, economic, and legislative factors in more than 100 countries has similarly revealed that the HDI has been strongly correlated with RTDR. Furthermore, considering the HDI components, education has been the most important dimension associated with RTDR, followed by income and LE.^6^ In this respect, a study in OECD countries from 2009 to 2018 confirmed that, even though the correlation between the HDI and road safety was unclear, developed countries encountered more opportunities to invest in their infrastructure, education, health care system, and improvement of road-user behavior. They also concluded that the role of socioeconomic factors was more vital in RTD in developing and least developed countries than in highly developed countries.^7^ Melinder investigated the relationship between religion and wealth in 15 Western European countries. They found that non-wealthy Catholic nations experienced more traffic accidents than wealthy nations, implying the importance of religion and wealth in RTD.^8^ Moreover, Bishai et al proposed four hypotheses about the relationship between economic growth and road casualties. First, more developed countries have a better institutional capacity to control externalities. Second, there is a competing risk story in which developing countries prefer reducing the risk of infectious and nutritional health risks to investing in road safety. Third, there is a vehicle mix story in which safer vehicles are used in affluent countries instead of high-risk transportation such as motorized bicycles and roofed buses. Finally, there is a medical technology story in which health care systems should be highly developed to deal with road trauma victims.^9^

 Different studies have addressed the relationship between income and RTDR. In 2003, Kopits and Copper examined 88 countries between 1963 and 1999. They reported that RTDR first increased, following a rise in income per capita, and then declined after reaching its peak.^10^ Another study in 2009 demonstrated a relationship between motorcycle fatality and economic growth in 25 countries from 1970 to 1999. The turning point reached a gross domestic product (GDP) per capita of US$ 12 682.^11^

 Relevant studies have mainly focused on a limited number of developed countries. Furthermore, to the best of the authors’ knowledge, they have not examined the association between the HDI components and RTDR in a time-trend analysis.^5-7^ As mentioned above, HDI is a comprehensive indicator showing the country’s level of development. Given the lack of longitudinal data about road safety, the present study focused on assessing the relationships between HDI and its components with RTDR to analyze the RTDR trends and their link with the HDI and its components between 2000 and 2019. The present study aimed to classify the countries based on the HDI and its components into downward or upward trends. The innovation of this study is first using the overall trend of RTDR from each country as the response variable in machine learning methods. Second, the rate of changes in HDI and its components were considered independent variables to model RTDR.

## Materials and Methods

###  Materials

 The dataset included RTDR and the HDI and its components from 154 countries between 2000 and 2019. The initial dataset comprised 183 countries, which was reduced to 154 cases after eliminating nations with missing data and those with a population of less than 1 million. The data on the target variable, RTDR, were collected from the WHO database.^12^ The data on covariates, HDI and its components, were further selected from the UNDP.^13^ The list of countries and their characteristics are given in Supplementary file 1.

###  Statistical Analysis

 The latent growth model (LGM) was used to assess the RTDR trends over 20 years. First, the unconditional linear LGM was utilized to identify the trajectory of RTDR.^14,15^ Second, the conditional linear LGM was applied to assess the impact of the HDI and its components on the trajectory of RTDR. Since the HDI and its components did not vary considerably over time, the mean values of the HDI and its components were considered time-invariant covariates. Third, the slopes from the unconditional linear LGM were used to determine the trends of RTDR in the countries concerned. Then, a binary dependent variable was defined as follows:

 0 = countries with a downward trend of RTDR and 1 = countries with an upward trend of RTDR. Fourth, the classification and regression trees (CARTs) were applied to identify the relationship between the defined binary variable and the HDI and its components.

**Figure 1:**
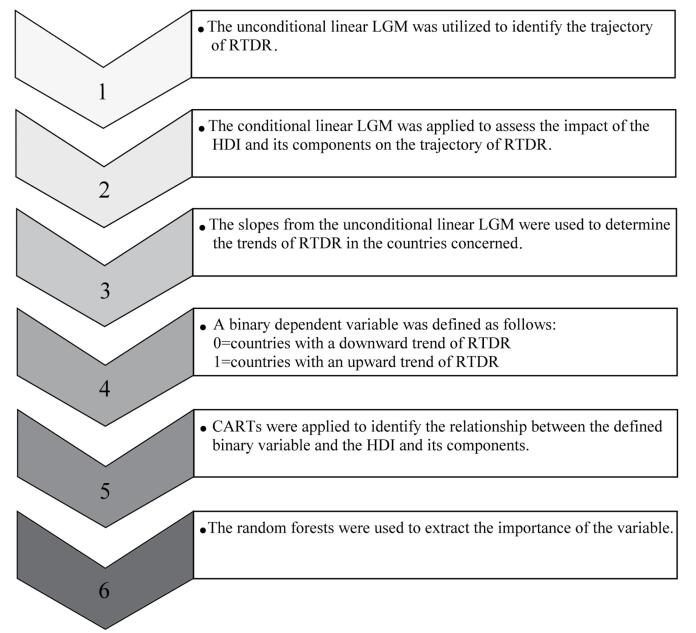
The Step-by-Step Process of Data Analysis. LGM: latent growth model, RDTR: road traffic death rate, HDI: human development index, CARTs: classification and regression trees

 Furthermore, to avoid the sensitivity of a single tree resulting from the CART models, random forests (RFs) were used to extract the importance of the variable.^16,17^ Moreover, 10-fold cross-validation was carried out to obtain the optimal CART and RF models. Additionally, the root mean square error of approximation (RMSEA) and the comparative fit index (CFI) were used to assess the goodness-of-fit (GoF) of the LGM models. Accordingly, the CFI values of greater than 0.95 indicated a good fit, while the RMSEA of less than 0.08 suggested a good fit.^15^ The significance level of the parameter estimations of the LGM was set at 0.05. Figure 1 demonstrates the step-by-step process of data analysis. The LGM was also performed using Mplus software version 7.0.^18^ The CART and RF procedures were carried out using rpart and random forest packages in the R statistical software version. 4.1.1.^19,20^

###  Latent Growth Model 

 The LGM could estimate the outcome growth trajectory by analyzing the development patterns of the data over time. This model comprised two growth parameters: the initial point (i.e., intercept) and the rate of changes over time (slope). The unconditional linear LGM is described as follows:


$$\begin{array}{l} y_{ti}=\eta_{0i}+\eta_{1i}\lambda_{t}+\varepsilon_{ti}\\ \eta_{0i}=\eta_{0}+\varsigma_{0i}\\ \eta_{1i}=\eta_{1}+\varsigma_{1i} \end{array}$$

where *y*_ti_ is the ith observed response measure at time point t, *η*_0i_ is the intercept component, *η*_1i_ is the linear slope component, *λ*_t_ are factor loadings, *η*_0_ denotes the estimated overall mean of the initial response, *η*_1 _represents the average rate of response change over time and *ε*_ti_, *ς*_0i_ and *ς*_1i_ are error terms. The LGM could also allow estimating the effect of covariates on latent growth parameters. The conditional LGM can be defined as follows:


$$\begin{array}{l} y_{ti}=\eta_{0i}+\eta_{1i}\lambda_{t}+\varepsilon_{ti}\\ \eta_{0i}=\eta_{0}+\sum_{k}\gamma_{0k}x_{ki}+\varsigma_{0i}\\ \eta_{1i}=\eta_{1}+\sum_{k}\gamma_{1k}x_{ki}+\varsigma_{1i} \end{array}$$

where *x *indicates the time-invariant covariates and *γ* are coefficients relating covariates to growth parameters.^21^ The path diagrams of unconditional and conditional LGM are illustrated in Supplementary file 2: Figure A1-A2.

###  Classification and Regression Trees 

 As a machine-learning procedure, CARTs were based on the nature of the dependent variable, which could be applied to classification and regression. This tree-based procedure aimed to partition the dataset into homogeneous subsets, namely terminal nodes, with regard to the dependent variable. Since the dependent variable was discrete (binary), the CARTs could minimize the Gini index as a criterion to create the final optimal tree.^22,23^ The Gini index is defined as follows:


$$Gini\left(m\right)=1-\sum_{j=1}^{J}P^{2}\left(j\left|m\right.\right)$$

With:


$$P\left(j\left|m\right.\right)=\frac{P\left(j,m\right)}{P\left(m\right)},\;P\left(j,m\right)=\frac{\pi \left(j\right)\;N_{j}\left(m\right)}{N_{j}}\;and\;P\left(m\right)=\sum_{j=1}^{J}P\left(j,m\right)$$

where *J* is the number of classes, *π(j)* is the prior probability of class *j*, *N*_j_*(m)* is the number of observations in class j of node m, *N*_j_ is the number of observations of class *j* in the root node, *P( j │ m )* is the estimated probability of an observation being in class j provided that it belongs to node m.^24^ The prediction performance of the classification trees was also assessed by accuracy, defined as follows:


$$Accuracy=\frac{sum\;of\;true\;classified\;cases}{total\;number\;of\;cases\;}\times 100$$

###  Random Forests

 RF is an aggregation of several CARTs.^16^ RFs could thus generate an ensemble of trees using bootstrap sampling and a randomized subset of predictors to enhance prediction performance.

## Results

###  Dataset 

 The common descriptive statistics for RTDR and HDI are presented in Table 1. As can be seen, RTDR ranged from 2.09 to 64.6 per 100 ­000 population over 20 years. According to the HDI analysis in 2019, among 154 countries, 56 cases were categorized as very high (HDI ≥ 0.8), 38 countries as high (0.7 ≤ HDI < 0.8), 28 cases as medium (0.55 ≤ HDI < 0.7), and 32 countries as low development (HDI < 0.55).^3^

**Table 1:** The Road Traffic Death Rate and Human Development Index of a Total of 154 Countries Between 2000 and 2019

		**Years**																			
		**2000**	**2001**	**2002**	**2003**	**2004**	**2005**	**2006**	**2007**	**2008**	**2009**	**2010**	**2011**	**2012**	**2013**	**2014**	**2015**	**2016**	**2017**	**2018**	**2019**
Road traffic death rate	Minimum	6.50	5.38	4.82	4.88	4.95	4.38	4.61	4.94	4.67	4.12	3.04	3.36	2.99	2.86	3.01	2.56	2.66	2.12	2.14	2.09
	Maximum	43.99	39.28	39.94	43.41	41.27	41.00	41.07	39.54	38.86	38.66	38.33	38.21	37.32	39.96	37.91	38.75	39.92	46.30	53.46	64.60
	Mean	19.73	19.66	19.83	19.88	19.80	19.64	19.78	19.65	19.34	18.76	18.35	18.13	17.89	17.69	17.57	17.64	17.57	17.54	17.47	17.57
	Standard deviation	8.09	7.97	8.22	8.55	8.46	8.47	8.66	8.36	8.38	8.61	8.76	8.77	8.99	9.16	9.11	9.24	9.57	10.10	10.31	10.91
Human development index	Minimum	0.262	0.268	0.273	0.276	0.285	0.294	0.300	0.306	0.314	0.321	0.331	0.338	0.350	0.357	0.365	0.372	0.378	0.386	0.391	0.394
	Maximum	0.915	0.914	0.917	0.923	0.932	0.931	0.934	0.936	0.937	0.937	0.941	0.942	0.944	0.946	0.944	0.947	0.950	0.954	0.956	0.957
	Mean	0.622	0.627	0.633	0.638	0.645	0.651	0.658	0.665	0.672	0.676	0.682	0.687	0.693	0.697	0.702	0.705	0.708	0.712	0.715	0.718
	Standard deviation	0.175	0.175	0.174	0.175	0.174	0.173	0.172	0.171	0.169	0.166	0.164	0.163	0.161	0.160	0.160	0.160	0.159	0.159	0.158	0.158

###  Unconditional Linear LGM

**Figure 2:**
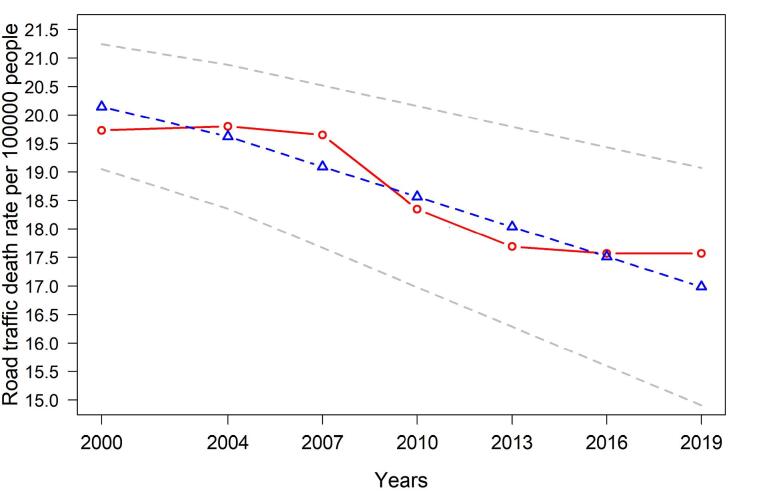
Observed Overall Trajectory (Red Solid Line) and Estimated Overall Trajectory (Blue Dashed Line) of Unconditional Linear LGM with 95% Confidence Interval (Grey Dashed Line) from 2000 to 2019

 The RMSEA and the CFI values were 0.035 and 0.976, respectively, representing an acceptable model fit. The estimated RTDR at the initial point was also 20.148 (*P* < 0.001). Besides, the significant negative slope (-0.527, *P* < 0.001) implied a decreasing trend in RTDR. Figure 2 shows the trajectories of the unconditional linear LGM.

###  Conditional LGM

 An initial analysis was performed to compare the performance of the LGM model with the HDI as a time-invariant covariate to the LGM model with the IHDI as a time-invariant covariate. The fit indices also showed that the LGM model with the HDI as a time-invariant covariate outperformed the LGM model with IHDI as a time-invariant covariate (Supplementary file 2: Table A1). Therefore, the mean value was considered a time-invariant covariate. The RMSEA and the CFI values were also equal to 0.032 and 0.984, respectively, suggesting an acceptable fit of the conditional LGM. The fit indices of conditional LGM indicate better performance compared to the unconditional LGM (Supplementary file 2: Table A2). The parameter estimations correspondingly implied the significant negative effect of the HDI on the intercept (-29.257, *P* < 0.001), denoting that countries with higher HDI had a lower initial value of RTDR. Moreover, the HDI had a significant negative effect on the slope (-3.438, *P* < 0.001), indicating a drop in RTDR associated with an upsurge in the HDI.

 To further investigate the role of the HDI in the trajectory of RTDR, the mean values of the HDI components were considered time-invariant covariates. Table 2 summarizes the results of the linear conditional LGM influenced by education, income, and LE. Since the mean of the HDI components were highly correlated (see multicollinearity problem, Supplementary file 2: Table A3), the effect of each component on the LGM parameters was reported separately. The results revealed the negative effect of education, income, and LE on the intercept and the slope. This implied that for example, education was negatively associated with RTDR in the baseline year 2000, and increased education was associated with decreased RTDR over 20 years. In addition, in order to observe the effect of collinearity of HDI components in LGM, the results of conditional LGM with education, income, and LE are represented in Supplementary file 2: Table A4. The results of the linear conditional LGMs with the HDI and its components as time-varying variables are also provided in Supplementary file 2: Table A5-A8.

**Table 2:** The Parameter Estimations of Linear Conditional LGM (HDI Component as a Time-invariant Covariate)

**Model (Time-Invariant Covariate)**	**CFI**	**RMSEA**	**Effect on Intercept **	**Effect on Slope **
Linear conditional LGM (Education)	0.971	0.023	-23.458^*^	-3.160^*^
Linear conditional LGM (Income)	0.969	0.040	-23.800*	-3.018*
Linear conditional LGM (Life expectancy)	0.957	0.036	-36.738*	-3.189*

CFI: comparative fit index; RMSEA: root mean square error of approximation. *Significant at 0.05 level.

###  Classification and Regression Trees 

**Figure 3:**
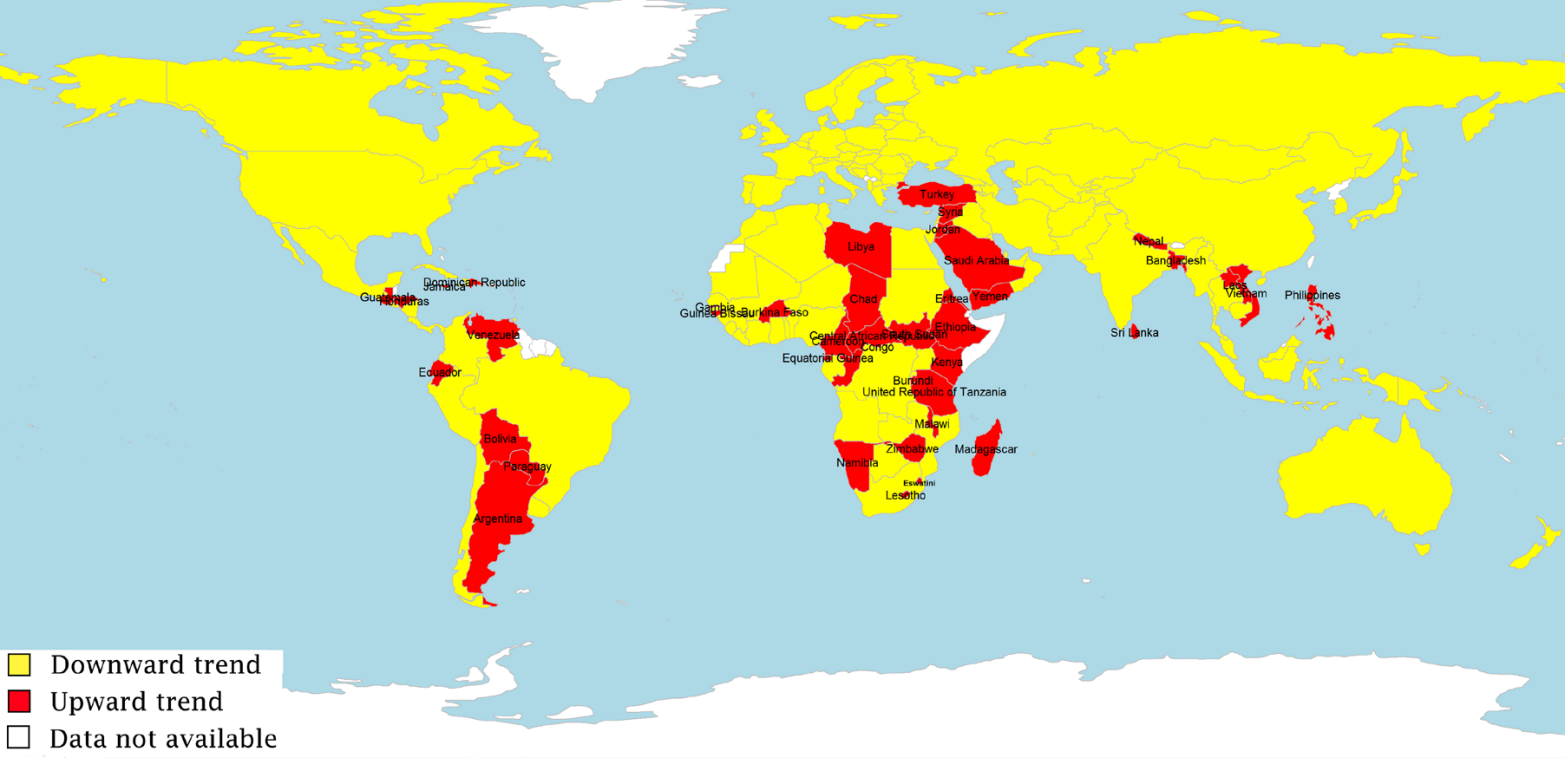
A Global View of Trends of RTDR from 2000 to 2019

 The estimated slope of RTDR for each country from the linear unconditional LGM was used for determining the overall trends of RTDR. Accordingly, 113 and 41 countries had a downward and upward trend of RTDR, respectively. Figure 3 shows the categorical world map based on the trends of RTDR using the rworldmap package in the R statistical software version 3.6.3.^25^ The estimated slope of the countries is provided in Supplementary file 1. The CART analysis was further performed to assess the relationship between the dependent binary variable and the HDI and its components. Since the CART procedure could choose the best splitter, the multicollinearity in the HDI components could be easily handled.^26^ Four models were also built using the CART procedure, that is, two models with the mean and the slope of the HDI as independent variables and the other two with the mean and the slope of the HDI components as independent variables.

**Figure 4:**
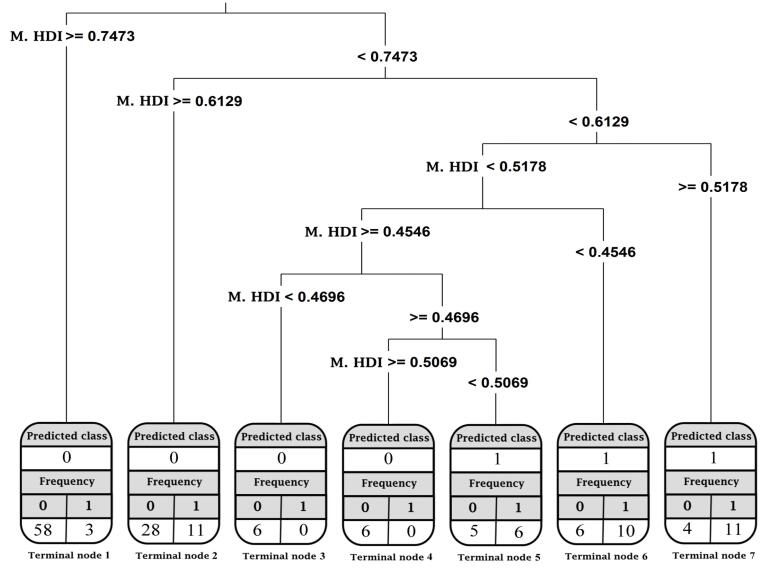
Optimal Tree Created by CARTs (Mean Values of HDI as Independent Variables). The predicted binary outcome and the number of countries in each category were shown at each terminal node. M. HDI: Mean value of the Human Development Index

Figure 4 displays the CART outcomes with the mean value of the HDI as an independent variable. The outcome was a tree with 7 terminal nodes. Additionally, 40% of the countries were placed in terminal node 1. The CART classified these countries in the downward category, and only three countries were misclassified, including Argentina, Saudi Arabia, and Libya. Moreover, 27% of the countries were classified in the upward category, with 15 misclassified countries. It is noteworthy that 29 countries were misclassified (having a different trend from the predicted class), which resulted in 81% accuracy.

**Figure 5:**
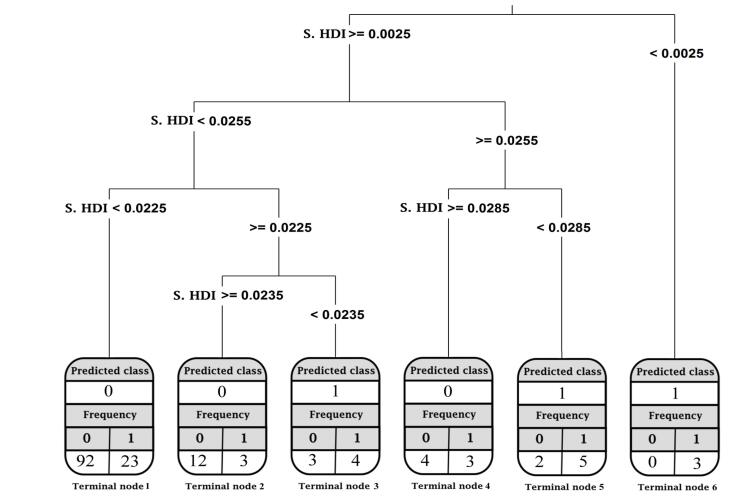
Optimal Tree Created by CART (Slope of HDI as Independent Variables). The predicted binary outcome and the number of countries in each category were displayed at each terminal node. S. HDI: Slope of Human Development Index

 Second, the CART result with an HDI slope as an independent variable is presented in Figure 5. These slopes were computed using the linear LGM. The outcome was a tree having 6 terminal nodes in which 75% of the countries were classified in node 1 (the slope of HDI between 0.0025 and 0.0225) as a downward category. Seventeen countries were also classified in the upward category, with 5 misclassified countries, including Guinea, Mozambique, Niger, Zambia, and Myanmar. Overall, the accuracy of the model was 78%.

**Figure 6:**
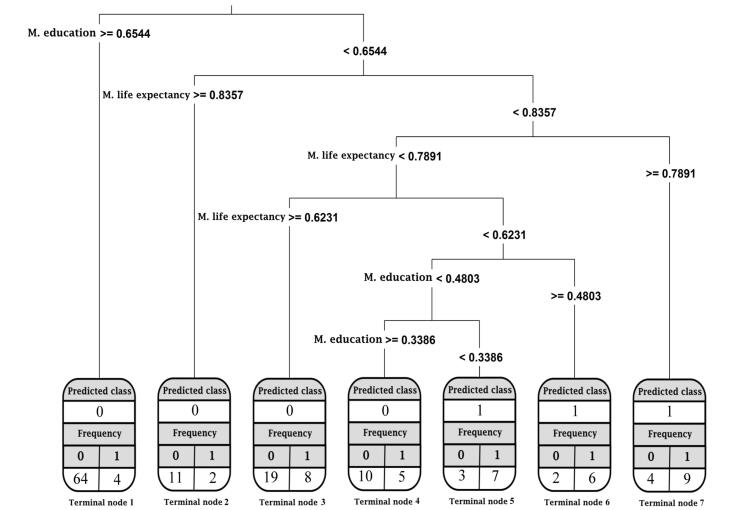
Optimal Tree Created by CART (Mean Values of Education, Income, and LE as Independent Variables). The predicted binary outcome and the number of countries in each category are shown in each terminal node. M. life expectancy: mean value of life expectancy, M. education: mean value of education, M. income: mean value of income

 Third, the outcome of the CARTs with the mean value of HDI components as independent variables was a tree comprised of 7 terminal nodes (Figure 6). It was observed that CARTs classified 44% of the countries assigned to terminal node one in the downward category. Furthermore, four countries were misclassified in this node (i.e., Saudi Arabia, Argentina, Sri Lanka, and Jordan). CARTs also predicted that the countries placed in the upward category in terminal nodes 5‒7, consisting of 20% of the countries, and nine countries were misclassified in these three nodes. Overall, 28 countries were misclassified. Therefore, the accuracy of the CART model with the mean value of the HDI components as independent variables was 82%, indicating the high classification performance of the model.

**Figure 7:**
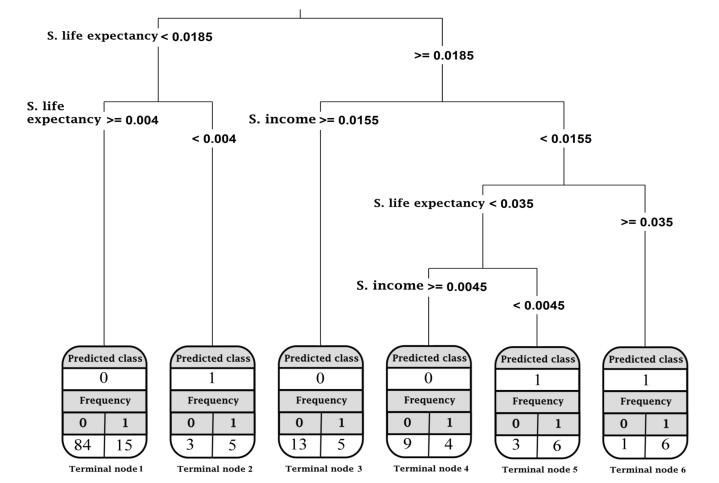
Optimal Tree Created by CART (Slope of Education, Income, and LE as Independent Variables). The predicted binary outcome and the number of countries in each category are shown in each terminal node. S. life expectancy: slope of life expectancy, S. education: slope of education, S. income: slope of income

Figure 7 illustrates the CART results with the slope of the HDI components as independent variables. These slopes were calculated using the linear LGM. The outcome was a tree made up of 6 terminal nodes. If the slope of LE of the countries ranged between 0.004 and 0.0185, the CART could allocate these countries to terminal node 1 (64% of all countries and those classified in the downward category). Moreover, the accuracy of the CART model with the slope of the HDI components as independent variables was 80%. The CART pruning rules, the mean value of RTDR, and the misclassified countries in each terminal node for all four models are provided in Supplementary file 2: Table A9-A12.

###  Variable Importance

 Variable importance measure, as one of the useful outputs of the tree-based models, could reflect the effect of the predictor variables on the model. The ranking of the variable importance in the RF model was more accurate than the CART.^27^ In this study, the independent variables from two CART models (with the mean and the slope of the HDI components) were integrated into the RF model to produce a more accurate ranking. Table 3 displays variable importance based on the increase in node purity measure. Education was the most important variable in the mean model, followed by LE. In the slope model, LE was the most critical variable, followed by income.

**Table 3:** Importance of Variables in Two CART Models Using Random Forest Procedure

**Variable**	**Increases in Node Purities**	
	**The Mean Value of the HDI Components Model**	**The Slope of the HDI Components Model**
Education	21.17	17.33
Life expectancy	19.94	20.67
Income	18.82	17.73

## Discussion

 This study revealed the decreasing global trends of RTDR in the studied period. Nevertheless, 41 countries out of 154 cases examined displayed increasing trends. According to the conditional LGM, the results indicated the negative effect of the HDI and its components on the intercept and the slope. Furthermore, education was the most important HDI component, negatively associated with RTDR in the mean model. In line with these results, Rahmanian Haghighi et al used two machine learning methods in a cross-sectional study to show that among the HDI components, education had the strongest association with RTDR. Moreover, our findings demonstrate that countries with lower slope changes in the HDI and LE mainly had a downward trend in RTDR. According to Ho and Hendi, the life expectancy of a country is a reflection of its social, economic, and quality of public health and healthcare infrastructure.^28^ Sirajudeen et al used life expectancy as an indicator to measure a country’s healthcare status and found a positive correlation between motorcycle deaths to passenger car deaths ratio and LE.^29^ In another study, Jamroz demonstrated the negative impact of LE on road fatality rate.^30^

 Most countries with an ascending trend in RTDR were in Africa, South America, or Southeast Asia. Among 154 countries, Qatar, South Africa, Lithuania, Latvia, Iran, and Russia have displayed the greatest reduction in RTDR between 2000 and 2019. On the other hand, South Sudan, Paraguay, Namibia, Guatemala, the Dominican Republic, and Saudi Arabia have faced the most increasing RTDR in this period. Besides, some countries had deviant behaviors compared with other nations in the same category. Among countries with very high HDI, Saudi Arabia, Turkey, and Argentina were the only cases experiencing a rise in RTDR in this period.

 Some studies have also assessed the relationship between the HDI and RTDR.^5,7^ Nevertheless, the relationship between the HDI components and RTDR has not been evaluated in a time-trend analysis. Considering the relationship between the mean value of different components of the HDI and RTDR, with the model accuracy of 82%, the mean of education was the essential factor associated with RTDR. Based on this model, the mean value of education, LE, and income influenced RTDR, respectively. Besides, the present study analyzed the relationship between the rate of various components of the HDI and RTDR. In this regard, the accuracy of the proposed model was 80%. The variable importance table indicates that the slopes of LE, income, and education were strongly associated with RTDR.

 Overall, the present study showed the significance of changing HDI, education, and LE in RTDR globally. Countries with an HDI value of more than 0.7473 or a change in their HDI slope between 0.0025 and 0.0225 could thus reduce RTDR between 2000 and 2019. Countries with a mean education of more than 0.6544 had mainly controlled RTDR, better than those with a lower index. As mentioned by the UNDP, there was a significant gap in education among countries with different human development categories. There is an additional 7.5 years of schooling for adults in nations with very high human development levels compared to countries with lower levels of human development, and an additional 7 years of schooling for children entering primary school in these countries.^3^ Moreover, nations with slighter changes in LE from 2000 to 2019 had a better association with reduced RTDR. Based on the hypothesis proposed by Bishai^9^ (mentioned earlier), it was concluded that countries with medium HDI had invested more in controlling health risks, such as infectious diseases and their nutritional status, than in improving road safety. Therefore, low- and middle-income countries should consider their limits and set realistic targets when developing their programs.

 The present study had several limitations. The main limitation was the lack of credible data at the global level (other than the HDI), which could help investigate its association with RTDR. For instance, data regarding road safety management, legislative factors, vehicle safety, and safer road users were not available for this time span. Hence, these variables were not considered in our analysis. Future studies should be conducted to provide a more comprehensive analysis of misclassified countries. Moreover, comparative studies between misclassified countries and countries with regular behavior with similar socioeconomic situations could be helpful for policymakers. On the other hand, in addition to highlighting the importance of HDI trend for predicting RTDR, the main strength of this study is that among HDI components, higher formal education and LE could contribute to RTDR reduction.

## Conclusion

 This study revealed the conflicting global trends of RTDR in the studied period, both decreasing and increasing trends were observed. The HDI and its components had negative effects on the intercept and the slope of global trends of RTDR, and among HDI components, higher formal education and LE could contribute to the reduction of RTDR. This finding may have implications for policymakers to reduce RTDR in their countries.

## Supplementary Files

Supplementary file 1. Countries’ characteristics and supplementary Data.

Supplementary file 2. Supplementary tables and figures.
