# Supplementary material for: The Global Road Traffic Death Rate and Human Development Index from 2000 to 2019: A Trend Analysis
Source: Arch Iran Med. 2024 Mar 1;27(3):113–21. doi: 10.34172/aim.2024.18 (PMC11097312; doi:10.34172/aim.2024.18)
Supplement: Supplementary file 1 — Countries’ characteristics and supplementary Data. [file aim-27-113-s001.pdf]

Arch Iran Med. March 2024;27(3):113-S1

10.34172/aim.2024.18

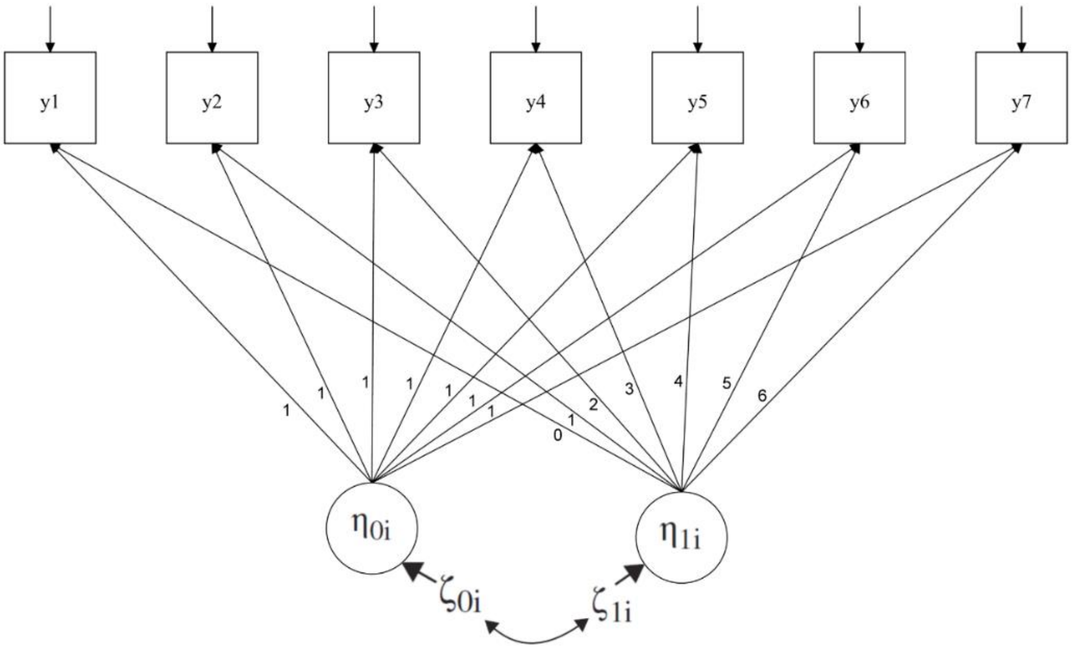

Figure A1. Path diagram of the unconditional latent growth model.

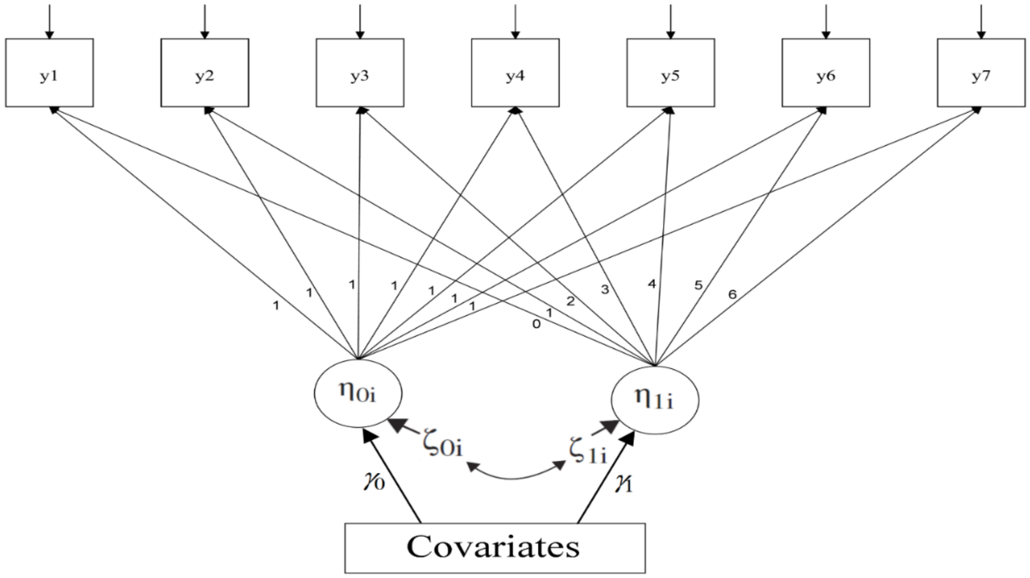

Figure A2. Path diagram of the conditional latent growth model.

Table A1. comparing the performance of the LGM model with HDI and LGM model with IHDI as time-invariant covariate.

Model	AIC	BIC	CFI	RMSEA
Linear conditional LGMM with HDI as time-invariant covariate	5707.50	5743.94	0.984	0.032
Linear conditional LGMM with IHDI as time-invariant covariate	5945.37	6100.04	0.977	0.043

Table A2. comparing the performance of the linear unconditional LGM and the LGM model with HDI as time-invariant covariate.

Model	AIC	BIC	CFI	RMSEA
Linear unconditional LGM	5875.91	6024.66	0.976	0.035
Linear conditional LGM with HDI as time-invariant covariate	5707.50	5743.94	0.984	0.032

Table A3. The correlations between HDI components.

Pearson Correlations			
	Mean life expectancy	Mean education	Mean income
Mean life expectancy	1	0.815**	0.839**
Mean education	0.815**	1	0.863**
Mean income	0.839**	0.863**	1
** Correlation is significant at the 0.01 level (2-tailed)			

Table A4. The parameter estimations of linear conditional LGM with mean of HDI components (education, income and life expectancy) as time-invariant covariates.

Model	education on intercept	education on slope	income on intercept	income on slope	life expectancy on intercept	life expectancy on slope
Linear conditional LGM	-2.435	-2.668*	2.322	-1.514	-36.663*	1.269 *

Table A5. The parameter estimations of linear conditional LGM (HDI as time-varying covariate).

	Estimate	S.E.	P-Value
HDI 2000 on road traffic death rate 2000	-22.917	2.616	0.000
HDI 2004 on road traffic death rate 2004	-26.517	2.461	0.000
HDI 2007 on road traffic death rate 2007	-30.179	2.412	0.000
HDI 2010 on road traffic death rate 2010	-35.867	2.394	0.000
HDI 2013 on road traffic death rate 2013	-40.177	2.524	0.000
HDI 2016 on road traffic death rate 2016	-43.920	2.677	0.000
HDI 2019 on road traffic death rate 2019	-47.460	2.966	0.000

Table A6. The parameter estimations of linear conditional LGM (education as time-varying covariate).

	Estimate	S.E.	P-Value
Education 2000 on road traffic death rate 2000	-14.453	2.854	0.000
Education 2004 on road traffic death rate 2004	-17.460	2.630	0.000
Education 2007 on road traffic death rate 2007	-20.636	2.618	0.000
Education 2010 on road traffic death rate 2010	-26.024	2.501	0.000
Education 2013 on road traffic death rate 2013	-29.820	2.572	0.000
Education 2016 on road traffic death rate 2016	-32.961	2.712	0.000
Education 2019 on road traffic death rate 2019	-35.968	2.953	0.000

Table A7. The parameter estimations of linear conditional LGM (income as time-varying covariate).

	Estimate	S.E.	P-Value
Income 2000on road traffic death rate 2000	-11.938	3.321	0.000
Income 2004 on road traffic death rate 2004	-14.591	3.246	0.000
Income 2007 on road traffic death rate 2007	-17.212	3.240	0.000
Income 2010 on road traffic death rate 2010	-22.018	3.349	0.000
Income 2013 on road traffic death rate 2013	-25.510	3.559	0.000
Income 2016 on road traffic death rate 2016	-28.456	3.779	0.000
Income 2019 on road traffic death rate 2019	-31.013	4.122	0.000

Table A8. The parameter estimations of linear conditional LGM (life expectancy as time-varying covariate).

	Estimate	S.E.	P-Value
Life expectancy 2000 on road traffic death rate 2000	-27.525	3.034	0.000
Life expectancy 2004 on road traffic death rate 2004	-31.926	3.066	0.000
Life expectancy 2007 on road traffic death rate 2007	-36.398	3.217	0.000
Life expectancy 2010 on road traffic death rate 2010	-42.314	3.618	0.000
Life expectancy 2013 on road traffic death rate 2013	-47.127	4.077	0.000
Life expectancy 2016 on road traffic death rate 2016	-51.303	4.587	0.000
Life expectancy 2019 on road traffic death rate 2019	-55.410	5.187	0.000

Table A9. CART pruning rules (mean of HDI as time-invariant covariates).

Terminal node	Rule	Predicted class	Number of countries at each class		Mean of mortality rate	Misclassified countries
			0	1		
1	If (M. HDI >= 0.7473)	0	58	3	12.11	Argentina, Saudi Arabia, Libya
2	If (0.6129 =< M. HDI < 0.7473)	0	28	11	19.34	Sri Lanka, Turkey, Venezuela, Jordan, Ecuador, Jamaica, Dominican Republic, Paraguay, Philippines, Bolivia, Viet Nam
3	If (0.4546 =< M. HDI < 0.4696)	0	6	0	27.32	-
4	If (0. 5069=< M. HDI < 0.5178)	0	6	0	20.67	-
5	If (0.4696=< M. HDI < 0.5069)	1	5	6	27.52	Nigeria, Benin, Uganda, Haiti, Cote d'Ivoire
6	If (M. HDI < 0.4546)	1	6	10	29.93	Democratic Republic of the Congo, Guinea, Mozambique, Sierra Leone, Mali, Niger
7	If (0.5178 =< M. HDI < 0.6129)	1	4	11	20.37	Timor-Leste, India, Ghana, Cambodia

M. HDI = mean of Human Development Index

Table A10. CART pruning rules (slope of HDI as time-invariant covariates).

Terminal node	Rule	Predicted class	Number of countries at each class		Mean of mortality rate	Misclassified countries
			0	1		
1	If (0.0025 =< S. HDI < 0.0225	0	92	23	16.74	Cameroon, Chad, Viet Nam, Guatemala, Ecuador, Lesotho, Dominican Republic, Guinea-Bissau, Sri Lanka, Bolivia, Venezuela, Paraguay, Argentina, Philippines, Gambia, Central African Republic, Madagascar, Honduras, Equatorial Guinea, Jamaica, Eritrea, Yemen, South Sudan
2	If (0.0235 =< S. HDI < 0.0255)	0	12	3	21.20	Bangladesh, Lao People's Democratic Republic, Kenya
3	If (0.0225=< S.HDI < 0.0235	1	3	4	28.47	Guinea, Mozambique, Niger
4	If (0.0285 =< S. HDI)	0	4	3	28.21	Zimbabwe, Eswatini, Ethiopia
5	If (0.0255 =< S. HDI < 0.0285)	1	2	5	23.12	Zambia, Myanmar
6	If (S.HDI<0.0025)	1	0	3	22.13	-

S. HDI = slope of Human Development Index

Table A11. CART pruning rules (mean of education, income and life expectancy as time-invariant covariates).

Terminal node	Rule	Predicted class	Number of countries at each class		Mean of mortality rate	Misclassified countries
			0	1		
1	If (M. education >= 0.6544)	0	64	4	12.57	Argentina, Sri Lanka, Saudi Arabia, Jordan
2	If (M. life expectancy >= 0.8357) & (M. education < 0.6544)	0	11	2	18.85	Ecuador, Viet Mam
3	If (0.6231<=M. life expectancy < 0.7891) & (M. education < 0.6544)	0	19	8	19.94	Bolivia, Philippines, Madagascar, Bangladesh, Nepal, Lao People's Democratic Republic, Yemen, Eritrea
4	If (M. life expectancy < 0.6231) & (0.3386=< M. education < 0.4803)	0	10	5	29.47	Cameroon, Equatorial Guinea, Malawi, Tanzania, Guinea-Bissau
5	If (M. life expectancy < 0.6231) & (M. education < 0.3386)	1	3	7	28.29	Guinea, Mali, Niger
6	If (M. life expectancy < 0.6231) & (0.4803<=M. education < 0.6544)	1	2	6	28.33	Botswana, Zambia
7	If (0.7891<=M. life expectancy < 0.8357) & (M. education <0.6544)	1	4	9	21.42	Brazil, Thailand, Nicaragua, Morocco

M. life expectancy = mean of life expectancy, M. education = mean of education

Table A12. CART pruning rules (slope of education, income and life expectancy as time-invariant covariates).

Terminal node	Rule	Predicted class	Number of countries at each class		Mean of mortality rate	Misclassified countries
			0	1		
1	If (.004=<S. life expectancy <0.0185)	0	84	15	14.98	Bangladesh, Sri Lanka, Dominican Republic, Paraguay, Nepal, Philippines, Argentina, Ecuador, Equatorial Guinea, Guatemala, Honduras, Saudi Arabia, Jordan, Gambia, Yemen
2	If (S. life expectancy < 0.004)	1	3	5	19.17	Cuba, United States of America, Mexico
3	If (S. life expectancy >=0.0185) & (S. income>= 0.0155)	0	13	5	23.91	Ethiopia, Zimbabwe, Lao People's Democratic Republic, Congo, Turkey
4	If (.0185=<S. life expectancy <0.0305) & (0.0045<=S. income< 0.0155)	0	9	4	25.75	Chad, Burkina Faso, Bolivia, Cameroon
5	If (0.0185=<S. life expectancy < 0.035) & (S. income <0.0045)	1	3	6	28.81	Cote d'Ivoire, Mali, Gabon
6	If (S. life expectancy>=0.035) & (S. income < 0.0155)	1	1	6	29.50	South Africa

S. life expectancy = slope of life expectancy, S. income = slope of income
